# A Single-Centre Retrospective Analysis of Pregnancies with Placenta Accreta Spectrum (PAS): From One-Step Surgery towards Two-Step Surgical Approach

**DOI:** 10.3390/jcm13113209

**Published:** 2024-05-30

**Authors:** Laura Weydandt, Massimiliano Lia, Amanda Schöne, Janine Hoffmann, Bahriye Aktas, Nadja Dornhöfer, Holger Stepan

**Affiliations:** 1 Department of Gynaecology, University Hospital Leipzig, 04103 Leipzig, Germany; bahriye.aktas@medizin.uni-leipzig.de (B.A.); nadja.dornhoefer@medizin.uni-leipzig.de (N.D.); 2 Department of Obstetrics, University Hospital Leipzig, 04103 Leipzig, Germany; massimiliano.lia@medizin.uni-leipzig.de (M.L.); holger.stepan@medizin.uni-leipzig.de (H.S.)

**Keywords:** placenta accreta spectrum, one-step surgery, two-step surgical approach, expectant management, blood loss, red blood cell units

## Abstract

**Background**: Placenta accreta spectrum (PAS) can be the cause of major morbidity and its optimal management is still controversial. The aim of this study was to compare the traditional one-step surgery with a two-step surgical approach in which the placenta is left in situ and the second final operation is delayed to minimise blood loss. **Methods**: We conducted a single-centre retrospective cohort study including all patients managed for PAS between 2007 and 2023. The number of units of red blood cells (RBCs) needed during surgery was the primary outcome used to compare these two approaches. **Results**: A total of 43 cases were included in this analysis. Twenty of these were managed with the delayed two-step surgical approach, whereas 23 received one-step surgery. The median estimated blood loss during surgery was 2000 mL and 2800 mL for two-step and one-step surgery, respectively (*p* = 0.095). In the two-step surgical approach, the median number of RBC units transfused during surgery was significantly lower (*p* = 0.049) and the odds ratio for needing more than four units of RBCs was 0.28 (95%-CI: 0.08–0.98, *p* = 0.043). A longer interval between the caesarean section and the second operation showed a trend toward lower blood loss (*p* = 0.065) and was associated with a significantly lower number of RBC units needed during surgery (*p* = 0.019). **Conclusions**: Two-step surgery for the treatment of PAS was safe in our cohort and could lead to a reduction in blood transfusion. Leaving the placenta in situ and delaying the final operation represents a possible alternative to traditional caesarean hysterectomy.

## 1. Introduction

Placenta accreta spectrum (PAS) still represents a cause of major morbidity and mortality in modern obstetrics. The incidence of this condition is estimated to be around 1:2500 pregnancies [1] and has been rising over the past decades according to population-based studies [2]. This development could be explained by the increasing rates of assisted reproductive technologies and caesarean sections, which are the strongest known risk factors for PAS [3].

There have been different approaches to managing PAS, the oldest one consisting of removing the placenta with the uterus en bloc during the primary caesarean section. This classic approach was first described 70 years ago [4] and has been approved by numerous publications and guidelines since then [5,6]. Throughout the years, different procedures were developed to avoid peripartum hysterectomy and its related morbidity and consequences. To date, four conservative procedures for managing placental abnormalities have been described in the literature: the extirpative technique, which involves the manual removal of the placenta; the expectant approach, characterised by leaving the placenta in situ; one-step conservative surgery, which entails removal of the accreta area; and the Triple-P procedure, involving suturing around the accreta area after resection [7]. Today, various centres treat patients with a uterus-preserving approach, leaving the placenta in situ during primary caesarean section and removing it later during a second surgery [8,9]. Expectant management is defined as leaving the placenta either partially or fully in situ and waiting for its spontaneous resorption or expulsion [10]. It must be mentioned that the aim of “expectant management” is not only the preservation of the uterus but also the reduction in blood loss and surgical morbidity.

Studies comparing conservative management with caesarean hysterectomy one-step surgery have shown different results and have been controversially discussed [7,11,12,13,14,15,16]. Recommendations vary among different guidelines, ranging from planned caesarean hysterectomy to leaving the placenta in situ with delayed removal as an option for women who desire to preserve their fertility [6,7].

Thus, the management of PAS has diversified and become more individualised in the last years.

The aim of this study was to describe the transition from one-step surgery (caesarean hysterectomy or conservative surgery immediately after caesarean section) to the two-step surgical approach (leaving the placenta in situ and removing it during a second surgical procedure) for treating PAS. This single-centre retrospective analysis compared patient outcomes between these two approaches.

## 2. Materials and Methods

We conducted a single-centre retrospective cohort study including all patients treated for PAS at the University Hospital of Leipzig between 2007 and 2023. This study was approved by the ethics committee of the medical faculty of the University of Leipzig (protocol code: 070/20-ek, date of approval 10 July 2020). In 2014, a two-step surgical approach was implemented at our institution and one-step surgery was abandoned. The aim of this study was to analyse its outcomes and to compare them to the historical cohort treated with traditional one-step surgery (either caesarean hysterectomy or extirpative technique). The primary outcomes were the number of units of red blood cells (RBCs) transfused during surgery and the estimated blood loss. Especially the number of RBC units transfused reflects PAS-related maternal morbidity as obstetrical blood estimation has been shown to be subjective in high volumes [17,18]. Secondary outcomes were the other complications during and after surgery. In the group managed with the two-step approach, the primary outcomes of the two operations (estimated blood loss and units of RBCs transfused) were added together.

### 2.1. Surgical Approaches and General Management of PAS

The one-step procedure (caesarean hysterectomy or conservative surgery immediately after caesarean section) was mostly abandoned in our centre in 2014 due to increasing evidence in favour of leaving the placenta in situ [19]. Consequently, a two-step surgical approach was first adopted at our institution in July 2014 and proposed to all patients with suspected PAS with the primary aim of reducing intraoperative blood loss. It consisted of leaving the placenta inside the uterus after a caesarean section followed by a second surgery where the placenta was removed either by hysterectomy, resection of the invaded area, or removal of the placenta without further surgical steps. The two-step approach has been consistently used since then at our institution with few exceptions. 

PAS was prenatally diagnosed using ultrasound using the sonographic marker described by Collins et al. [20]. When PAS was strongly suspected in antenatal imaging (ultrasound or MRI), a caesarean section was planned around the 35th week of gestation. In both the one-step and two-step surgical approach, the caesarean section was performed by midline incision, which was extended cranially above the umbilicus if necessary. The diagnosis of PAS was confirmed intraoperatively by the presence of (a) purple colouring and distension over the placental bed (b) significant hypervascularity running in the uterine serosa (c) placental tissue invading through the surface of the uterus with or without the involvement of other pelvic organs. In the case of hysterectomy, PAS was confirmed histopathologically and further classified postoperatively.

The newborn was delivered through a vertical fundal incision and oxytocin was avoided. In the two-step surgical approach, the umbilical cord stump was secured with non-resorbable thread, and the placenta was left in situ without any manipulation. The uterotomy was then closed with multiple layers of single sutures.

In the two-step surgical approach, patients systematically received antibiotics for one week after the first operation and laboratory examinations (fibrinogen, leucocyte count, and measurement of C-reactive protein) were performed every week at the beginning, then later only every two weeks. The timing of the second operation was generally delayed as long as tolerable for the patient. If relevant vaginal bleeding occurred, the operation was done within 24 h. Preservation of the uterus was generally preferred if deemed possible by the senior obstetrician/gynaecologist performing the operation. This decision was based on the extent of the placental involution and persistent parametrial invasion. Resection of the uterus (delayed hysterectomy) was also performed if the patient preferred this operation instead of uterine preservation. Methotrexate and uterine embolization were not used as these adjunctive treatments are currently not recommended [6,7].

Before 2014, PAS was managed by a one-step surgery encompassing the removal of the whole placenta after delivery and was accomplished by either caesarean hysterectomy, removal of the accrete area, or the extirpative technique [21]. The choice between these two procedures was at the discretion of the senior obstetrician/gynaecologist performing the operation and based on the respectability of the adherent uterine wall and the extent of the parametrial invasion. 

The surgical teams varied over time but always included an experienced senior obstetrician and an experienced senior gynaecologic surgeon. The choice of the surgical approach was left to the discretion of the experienced gynaecologic surgeon and was based on the clinical picture and the patients’ preferences expressed before surgery. The clinical diagnosis of suspected PAS was made based on the criteria described above.

The decision to perform intraoperative transfusion of RBCs was at the discretion of the anaesthesiologist in charge and, as recommended by national guidelines, based on the observed intraoperative blood loss and intraoperative haemoglobin levels [22]. Autologous cell-saver technology was occasionally used in both one-step and two-step surgical approaches (Table 1).

### 2.2. Data Collection and Preparation

The medical database of the University Hospital of Leipzig was systematically searched in order to select all patients treated for PAS between 2007 and 2023. PAS had to be confirmed either intraoperatively or histologically and cases where sonographically suspected PAS could not be confirmed were excluded from the cohort. We extracted the following data from medical records for statistical analysis: age, body mass index (BMI), parity, risk factors for PAS (number of caesarean sections or dilatation and curettage), usage of cell saver, estimated blood loss, number of RBC units used during surgery, intra- and postoperative complications, and hysterectomy rates. Histological reports of those patients with hysterectomy or resection of the accrete area were reviewed for the degree of placental invasion (accreta, increta, percreta). Patients were excluded from this study when a caesarean section was performed due to chorioamnionitis [23], as this condition could urge the surgeon to perform the one-step approach thus leading to an allocation bias. In those patients with conservative management, laboratory results (i.e., leucocyte count and C-reactive protein (CRP)) measured between the two surgeries were also collected.

### 2.3. Statistical Analysis

The Wilcoxon rank sum test was used to compare the primary outcomes (number of RBCs transfused and intraoperative blood loss) between the two study groups (one- and two-step procedures). 

As the surgical procedures within the study groups were different in nature (hysterectomy or removal of placenta with or without a part of the uterine wall) we additionally performed an analysis comparing the primary outcomes of the subgroups with hysterectomy and the subgroups managed without hysterectomy. Logistic regression was used to explore possible risk factors for the need for blood transfusions. The association between the number of days between the two operations (two-step group) and intraoperative blood loss or units transfused were analysed by linear regression. Furthermore, we used linear regression in order to examine a possible change in the primary outcomes over time separately for both study groups. This was done in order to evaluate if an improvement in the intraoperative blood loss or the number of RBC units transfused could also be explained by a learning curve rather than the change in the management of PAS. 

Other patient characteristics were compared using the Wilcoxon rank sum test (continuous variables) or Fisher’s exact test (categorical variables).

The *p*-values of <0.05 and <0.1 were regarded as significant and borderline significant, respectively. The statistical software package R (Version 4.1.0) was used for statistical computing and graphics.

## 3. Results

**Figure 1:**
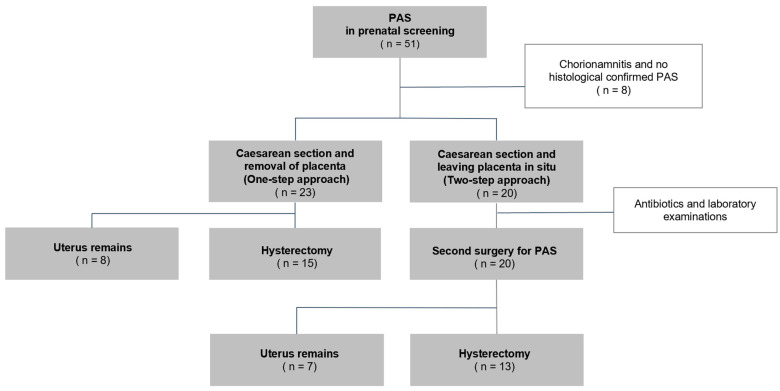
Overview: one-step surgical vs. two-step surgical approach resulting in hysterectomy or preservation of the uterus.

A total of 51 cases of PAS were managed at the University Hospital of Leipzig in the time period between 2007 and 2023. Seven cases were excluded from this analysis because of chorioamnionitis, which can naturally be associated with increased blood loss. Additionally, one case was excluded as PAS was not confirmed histologically. Thus, a total of 43 cases with PAS were included in the analysis (Figure 1). A total of 20 (46.5%) of these were managed with the two-step surgical approach, whereas 23 (53.5%) received one-step surgery. Hysterectomy could be avoided in eight (34.8%) cases in the one-step surgery group and in seven (35%) cases in the delayed two-step group. Planed hysterectomy upfront surgery at the patient’s request occurred in six cases (30%) in the one-step surgery group and in ten cases (43.5%) in the two-step approach. Intraoperative complications (ureter or bladder injury) were similar in both groups (Table 1). The median time between first and second surgery was 48 days (IQR 40.8–65) in the two-step approach. In the two-step surgery group, eight women (40%) had recurrent or relevant bleeding leading to hospitalisation. However, the rate of emergency operations (within 24 h) did not differ significantly (*p* = 0.3) between the one-step and the two-step surgery groups. In the whole cohort, the median intraoperative blood loss during emergency operation (3100 mL; IQR 2000–5875 mL) was not significantly different from the blood loss during planned operation (1900 mL; IQR 975–4250 mL; *p* = 0.11). No clinically relevant infection or sepsis were observed in the group managed by the two-step approach. 

**Figure 2:**
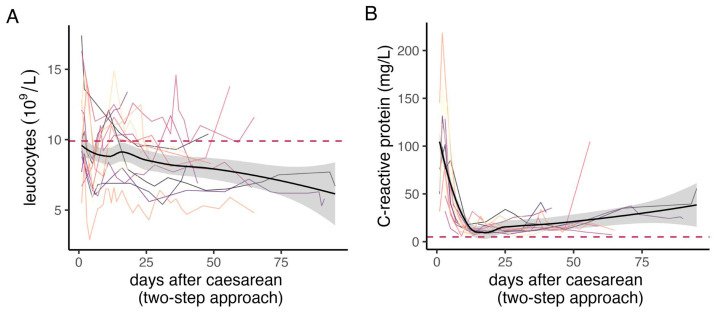
Inflammation parameters between surgeries in the two-step surgery group: (**A**,**B**) showing longitudinal values of CRP and leucocytes between operations in patients treated with the two-step approach. Each colour represents an individual patient, whereas the black line is the regression line with the confidence interval in grey. The red dashed line represents the upper limit of the reference value.

In the entire cohort, the median estimated blood loss was 2000 mL (IQR 1100–4800 mL), and 65.1% of the patients received blood transfusion at some point during the operations. The median number of RBC units transfused in the whole cohort was three (IQR 0–7), and 46.5% of patients received more than four units. A total of 20 patients (46.5%) did not need any intensive care management after the first or second surgery. In 30 cases, a definitive histological diagnosis could be made, and placenta accreta, increta, and percreta were reported in 36.7%, 26.6%, and 36.7%, respectively. In 13 cases, the grade of abnormal placental invasion could not be determined in histological analysis. Of note, gestational age at delivery in patients managed by two-step surgery was significantly higher than in those with the one-step approach (Table 1). The time period of both leucocytes and C-reactive-protein (CRP) between the two operations in the two-step approach are shown in Figure 2. Of note, CRP values after the first operation are usually high and fall sharply within the first two weeks but generally do not reach baseline levels until after the second surgery.

Median estimated blood loss during surgery was 2000 mL and 2800 mL for two- and one-step surgery, respectively (*p* = 0.095). The number of RBC units transfused was significantly lower in the two-step surgical approach (*p* = 0.049, Table 1) and the odds ratio of needing more than four units was 0.28 (95%-CI: 0.08–0.98, *p* = 0.043). 

**Figure 3:**
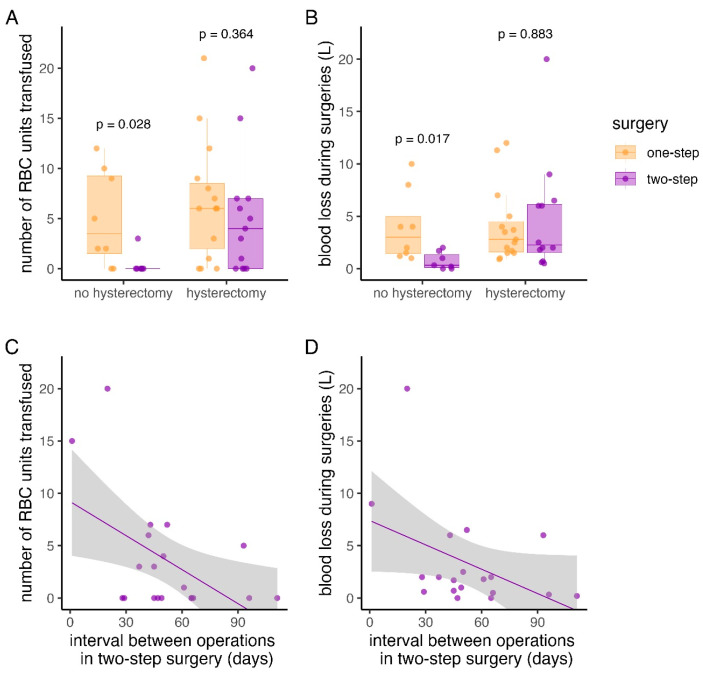
RBC units and blood loss: (**A**,**B**): number of RBC units transfused and blood loss during surgeries in patients with one-step surgery and the two-step surgical approach with box-plots. (**C**,**D**): linear regression of intraoperative estimated blood loss (*p* = 0.065) and number of transfused RBC units (*p* = 0.02) depending on the number of days between operations.

In particular, the subgroup without need for a hysterectomy had a significantly lower median number of RBC units transfused (0 units vs. 4 units; *p* = 0.028) and significantly lower median blood loss (330 mL vs. 3000 mL; *p* = 0.017) when the two-step surgical approach was performed (Figure 3A,B). However, when a hysterectomy was performed, neither the median number of RBC units transfused (4 units vs. 6 units; *p* = 0.88) nor the median blood loss (2250 mL vs. 2800 mL; *p* = 0.36) were significantly lower in the two-step surgical approach (Figure 3A,B). 

Univariable logistic regression was used to explore possible factors associated with the overall need for intraoperative transfusion of RBCs in the whole cohort. There was no statistically significant association between the need for transfusion and the number of caesarean sections (*p* = 0.70), completed gestational weeks at birth (*p* = 0.86), and patient’s age (*p* = 0.59) or BMI (*p* = 0.96). Only the type of management (two-step compared to one-step surgery) showed a trend to lower rates of blood transfusion in two-step surgery (OR 0.28; 95%-CI: 0.074–1.04; *p* = 0.057). This result did not change relevantly (OR 0.22; 95%-CI: 0.051–1.01; *p* = 0.051) after adjustment for the gestational week at birth, which was the only parameter that differed significantly between the two surgical approaches (Table 1).

Linear regression showed that longer intervals between the caesarean section and the second operation were associated with lower estimated blood loss (*p* = 0.065) and RBC units transfused (*p* = 0.019) in patients treated with the two-step approach. Specifically, for every additional day between the two surgeries, linear regression predicted a decrease in blood loss by 77 mL and a decrease in RBCs by 0.11 units (Figure 3C,D).

## 4. Discussion

This study shows that a two-step surgical approach in the treatment of PAS is associated with a lower blood loss and less units of RBCs transfused during surgery compared to the traditional one-step surgical approach. This benefit was especially pronounced when hysterectomy was not needed for the removal of the morbidly adherent placenta (Figure 3A,B). Additionally, we show the development of leucocyte and CRP levels during the period in which the placenta is left in situ. To the best of our knowledge, this is the first study showing that both leucocytes and CRP may be elevated when the placenta is left in situ without resulting in infection or sepsis.

Caesarean hysterectomy has traditionally been the recommended treatment in these cases [6], but other treatment options have been explored over the decades [7,15]. Several studies have demonstrated the advantages of leaving the placenta in situ including reductions in the rate of hysterectomy [16,24,25], blood loss [16,25,26], transfusion of blood products [16,24,27], cystotomy [26], and disseminated intravascular coagulation [24]. In a multicentre retrospective study including 167 cases of PAS managed by leaving the placenta in situ, the rate of serious maternal morbidity was 6% and one maternal death (0.6%) was observed [28]. Another study compared immediate hysterectomy to delayed hysterectomy in patients with placenta percreta over a time period of 6.5 years and observed lower blood loss and blood product transfusion [27].

In light of these results, the FIGO published a consensus guideline stating that leaving the placenta in situ is an option in women affected by PAS [7]. However, the American College of Obstetricians and Gynaecologists argues that conservative or expectant management should only be considered for carefully selected patients [6] and among clinicians and researchers this approach is controversially discussed [29,30].

The PACCRETA study [16] showed that the rate of transfusion of more than four units of RBCs was significantly lower in patients who received conservative treatment of PAS (OR 0.29). Interestingly, this study showed a very similar reduction when the two-step surgical approach was adopted (OR 0.28, 95%-CI: 0.063–1.15, *p* = 0.067). On a sidenote, the management strategy in the PACCRETA study did not involve a planned second surgery but rather leaving the placenta in situ to reabsorb.

The results of our study challenge those studies claiming a high rate of complications among patients where the placenta is left in situ. Importantly, these studies are literature reviews and base their conclusions on (at least partly) the same cases published in the literature [11,12,13]. Thus, the evidence arguing against leaving the placenta in situ may be overrated.

However, our study has several important limitations and influencing factors, which need to be addressed. One limitation of this study is the retrospective design with inherent limitations. Allocation of the patient to either two-step or one-step surgery may have been influenced by the obstetrician or the preferences of the women. However, after 2014, the two-step surgical approach was used rather consistently over the following years (79.2% after 2014 vs. 0% prior to 2014). Additionally, in the group managed by two-step surgery, hysterectomy was planned in a relevant part of women (30%), already before the second operation. We cannot determine retrospectively if this decision was based on the woman’s preferences or the finding during the first operation (i.e., caesarean section). 

Furthermore, the lower rate of transfusions in the two-step approach may have been influenced by other factors. Firstly, autologous cell-saver technology can decrease the number of RBC units needed during the operation. However, the fraction of cases in which this technology was used during surgery was similar in both subgroups (Table 1).

Secondly, the gestational week at delivery was significantly lower in the group with the one-step surgical approach, thus making confounding possible. However, after statistical adjustment for the gestational week, the rate of blood transfusion in the two-step approach remained constant (OR 0.28 compared to 0.22 when adjusted for gestational age) making it unlikely that the lower gestational week in the one-step approach was responsible for the higher rate of transfusions in this group. 

Thirdly, there may have been a learning curve in the management of PAS over time, explaining the improvement regarding blood loss and transfusion. Also, changes in the personnel could have influenced the intraoperative management and the decision to perform transfusion of RBCs. However, this is unlikely the case as blood loss and the number of RBCs transfused was constant over time in the one-step and two-step approach (Figure S1). 

Fourthly, we cannot be sure that severe cases of PAS were more common in one of the subgroups, as histological classification can only be done if an adequate specimen is available. Retrospective clinical classification based on surgical reports would be imprecise, as some operations happened before appropriate guidelines were published [21]. However, most cases did have histological reports and no significant difference was observed regarding the grades of PAS.

Lastly, the small sample size represents an important limitation. This study was conducted over a 16-year period and included only 43 patients (2–3 cases per year). However, this relatively small number of patients is comparable to those treated in other European centres. In a French multicentre study, the largest number of cases treated for PAS was 46 over the time span of 14 years [28]. In the PACCRETA study, 176 hospitals treated 253 patients with PAS in 2 years, less than one case per hospital per year [16].

Nevertheless, our results support the assumption that the two-step surgical approach is reasonably safe, additionally to its benefits in terms of blood loss and transfusion quantity. Of note, leaving the placenta in situ did not increase risk over time, as evidenced by a lower number of RBC units transfused when the interval between surgeries was longer, as the number of RBC units transfused was lower if the interval between surgeries was longer (Figure 3C,D). However, some women will experience relevant vaginal bleeding leading to an urgent surgical procedure. Consequently, women with PAS managed by the two-step surgical approach need to be able to urgently consult an appropriate hospital, which may be problematic in sparsely populated regions. Additionally, this approach comes at the cost of a second surgical procedure with additional anaesthesia and hospital stay. However, in a number of cases, this approach has led to preserved fertility with the option of a successful subsequent pregnancy. 

In this study, we could only analyse parameters with precise documentation (i.e., blood loss, transfusion, and intraoperative complications). Thus, more subtle but equally relevant outcomes, such as the psychological strain of a second surgery and hospitalisation, could not be examined due to the retrospective nature of this study. Consequently, future research needs to prospectively measure quality of life of patients when comparing therapies of PAS. 

## 5. Conclusions

This study suggests that the two-step surgery approach (leaving the placenta in situ with delayed second surgical management) is effective in reducing blood loss and the number of RBC units transfused. This benefit was especially pronounced when hysterectomy was not needed to remove the placenta during the second operation. Additionally, this approach was comparably safe as the one-step surgical approach without any differences in terms of emergency procedures. Furthermore, a longer interval between the two operations did not increase blood loss and number of RBC units needed during the second operation. Therefore, the individualised two-step surgical approach can be offered to patients as a feasible alternative to removing a morbidly adherent placenta immediately after the delivery of the newborn.

## Figures and Tables

**Table 1:** Patient’s characteristics. Continuous variables are expressed as medians and interquartile ranges. Two-sided Wilcoxon rank sum test and Fisher’s exact test were used to test for significance.

Overview of Patients’ Characteristics			
	Two-Step Surgical Approach (*n* = 20)	One-Step Surgical Approach (*n* = 23)	*p*-Value
Age (years)	34 (30.8–35)	31 (30–36.5)	0.9
BMI (kg/m^2^)	24 (20.7–31.1)	23.3 (21.1–27.1)	0.7
Height (cm)	165 (162–168)	165 (164–168)	0.75
Parity	2 (2–3)	2 (1–3)	0.34
Number of prior caesarean sections	2 (1–3)	1 (1–3)	0.65
Completed weeks of gestation at birth	35 (35–35.3)	33 (30–34.5)	0.009
Histological analysis performed (%)	12 (60.0)	18 (78.3)	0.2
Placenta accreta (%)	5 (41.7)	6 (33.3)	0.2
Placenta increta (%)	2 (16.7)	6 (33.3)	
Placenta percreta (%)	5 (41.7)	6 (33.3)	
Estimated blood loss (mL)	2000 (675–6100)	2800 (1500–4500)	0.095
Intraoperative blood transfusion (%)	10 (50)	18 (78.3)	0.064
Number of red blood cell units transfused during surgery	3 (0–7)	6 (2–9)	0.049
Transfusion of more than 4 units of RBCs (%)	6 (30)	14 (60.9)	0.043
Usage of autologous cell-saver technology (%)	5 (25)	5 (21.7)	1
Ureter injury (%)	2 (10)	2 (8.7)	1
Bladder injury (%)	2 (10)	3 (13)	1
Hysterectomy (%)	13 (65)	15 (65.2)	>0.9
Planed hysterectomy upfront (%)	6 (30)	10 (43.5)	0.4
Time between first and second surgery (days)	48 (40.8–65)		
Vaginal bleeding between surgeries leading to hospitalisation (%)	8 (40)		
Emergency operation * (%)	3 (15)	7 (30.4)	0.3
No admission to intensive care (%)	12 (60)	8 (34.8)	0.14
Admission to intensive care for one day (%)	8 (40)	13 (56.5)	
Admission to intensive care for two days (%)	0 (0)	2 (8.7)	

* Operation done within 24 h after the onset of relevant vaginal bleeding.

## Data Availability

The data presented in this study are available upon reasonable request. The data are not publicly available due to privacy and ethical reasons.

## Supplementary Materials

The following supporting information can be downloaded at: https://www.mdpi.com/article/10.3390/jcm13113209/s1, Figure S1: Blood loss and number of RBC transfused over time in the one-step and two-step approach.

## Abbreviations

PAS	Placenta accrete spectrum
BMI	Body mass index
RBC	Red blood cell
CRP	C-reactive-protein
ICU	Intensive care unit
