# Supplementary material for: A Single-Centre Retrospective Analysis of Pregnancies with Placenta Accreta Spectrum (PAS): From One-Step Surgery towards Two-Step Surgical Approach
Source: J Clin Med. 2024 May 30;13(11):3209. doi: 10.3390/jcm13113209 (PMC11172444; doi:10.3390/jcm13113209)
Supplement: Supplementary file 1 [file jcm-13-03209-s001.zip › jcm-3026149-supplementary.pdf]

Supplementary materials

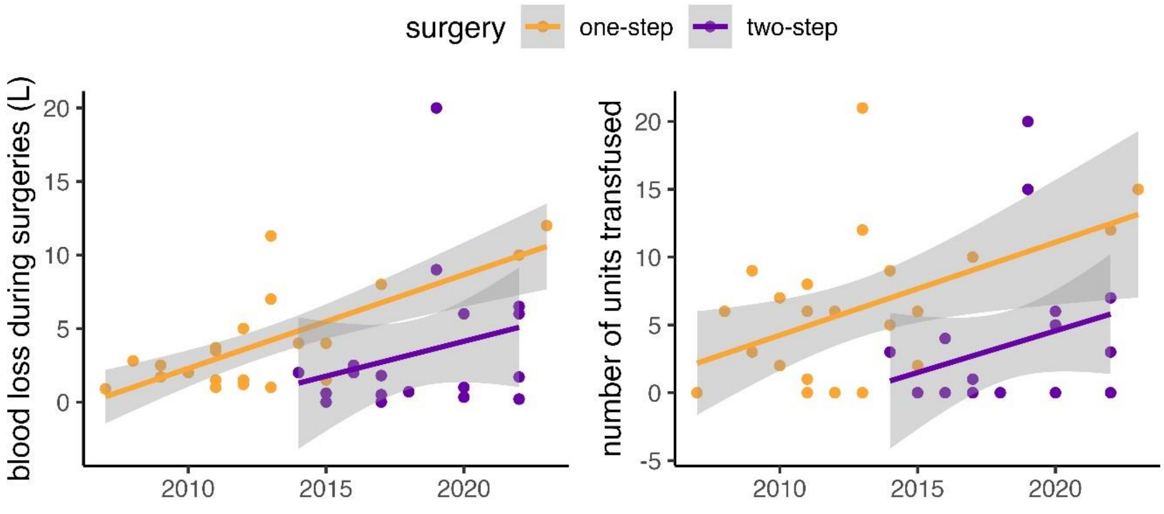

Figure S1. Blood loss and number of RBC transfused over time in the one-step and two-step approach.
